# Drug Powdering

**Published:** 1867-02

**Authors:** E. R. Squibb

**Affiliations:** Brooklyn


### Miscellaneous.

### Drug Powdering.

Substances which are used as medicines require in powdering more honesty, knowledge, care and attention, and a better and more cleanly apparatus than they generally get at the hands of the ordinary drug millers; and physicians, pharmaceutists and druggists should know this, and be much more careful than they are in the character and quality of their powdered drugs. To the ordinary drug mill all that comes is so much grist to be ground. Good and bad all go through alike, and pay the same profit. The tons of liquorice root for the tobacco manufacturer, and the dose of ergot upon which a human life often depends, are treated in the same way. The lot of hard, poor lumps of opium, picked out from the softer, richer portions, because they lose less in powdering, is powdered either alone or with any diluting substance that may be sent with it, and is considered to involve no responsibility to the miller, and really affects his interest only favorably in consequence of being easier to powder, and making a handsomer, more lively powder. But this handsome powder when put into the market a little under price, attracts the buyers, and leaves him who obtains a fair average quality for his powdered opium by sending the entire case, rich and poor, together, to be powdered, an unsuccessful salesman, though offering at a smaller profit. The kind of competition shown by this example is particularly fostered by drug mills which quietly grind all that is presented, because when a drug is powdered, the landmarks of condition and quality are all swept away, leaving very little except the bare statements of the salesman. His reliability, or want of it, must be accepted in all its broad uncertainty. There is now, and always must be, an open recognized market for all classes and qualities of drugs and medicines, and the inferior and bad will be bought, sold and used, as long as they yield better profits at lower prices. But the success which attends the market for low grades of medicines must—except for the drug mill—always depend upon cupidity or ignorance, since no one would buy a bad medicine rather than good, if both yielded the same profit, and if he knew the good from the bad; and since, in the crude state of drugs, there are few buyers or dealers who do not know the difference in appearance between the good and bad. Just here, however, the drug mill comes in, and while performing the indispensable duty of converting crude material into a condi tion for use, so changes the appearance that distinctions in quality may be entirely obliterated or even reversed, and the lower qualities may be made to appear and sell the best. Cupidity and ignorance are dangerous to all mercantile interests, but particularly so when, as in the case of medicines, they must be encountered unseen. The practical application of all this is, that he who buys powdered drugs with the right motive, should be sure of the salesman, since he can in no way be sure of anything else. There appear to be few positions in life where a man's word is of so much importance, and where it can be of so great a pecuniary value and so little intrinsic worth. Dealers in medicines fall into orders and classes by natural laws, just as everything in the great scheme of nature does. All in a short time acquire a commercial standing, and are rated accordingly upon the mercantile agency books, and in the channels of trade. But there is beside this a moral standing not so quickly or so easily acquired, though even more easily damaged and lost, and for which there is no mercantile agency. These different standings have no necessary relations, and in their highest degree, perhaps never coincide, and therefore buying powdered drugs on commercial standing alone, is, as a general thing, worse than buying at random. The moral status of the drug trade, including drug powdering, is not lower than that of other trades in general, and is even perhaps above that of a majority of them. But in it the importauce of a high moral standing is infinitely greater than in any other trade, because its results are vital. It is an odious task to go about like a scavenger, raking out the moral filth from the channels of trade and setting it in heaps which may appeal to the senses of the passers by; yet this must be done, and since the authority of law cannot be obtained either to prevent or remove the accumulations, those who do not like it at their doors must remove it themselves. There are those in all parts of the country who keep their paths clean, and there is integrity and industry enough to seek them out, and knowledge and discrimination enough to find them—and they are often found. But pecuniary interests, want of knowledge, and thoughtless inattention still so obstruct the way and increase the difficulties of the search, that the great mass of physicians are very badly served, and the bills of mortality swallow up the results unrecognized, as they do many other sugar-coated pills of error and effrontery.

The desire for pecuniary profit, and the importance of this subject, induce the undersigned, after many years' experience in a small way, to extend his business as a drug powderer, promising those who choose to employ his mills, that there shall be no mixing or adulteration practiced in them, nor in the material sent to them, and that the drugs shall be managed with due knowledge and respect for their character as medicines. This, however, can not be done at the low prices charged by other mills, because the profits must remain as good as theirs, whilst a more expensive apparatus, more labor, care and attention, and a smaller business, involve greater cost. E. R. Squibb.

*Brooklyn*, Nov. 20, 1866.

